# Ophthalmic Manifestations in Acute Leukemia Patients and Their Relation With Hematological Parameters in a Tertiary Care Center

**DOI:** 10.7759/cureus.19384

**Published:** 2021-11-08

**Authors:** Ziad M Bukhari, Abdulmalek Alzahrani, Mohammed S Alqarni, Rakan S Alajmi, Ali Alzahrani, Hashim Almarzouki, Abdullah S Alqahtani

**Affiliations:** 1 Medicine, King Abdullah International Medical Research Center, Jeddah, SAU; 2 Medicine, King Saud bin Abdulaziz University for Health Sciences, Jeddah, SAU; 3 Ophthalmology, King Abdullah International Medical Research Center, Jeddah, SAU; 4 Ophthalmology, King Abdullah Medical Complex, King Saud bin Abdulaziz University for Health Sciences, Jeddah, SAU

**Keywords:** hematological parameters, chemotherapy, retinal hemorrhage, acute leukemia, ophthalmic manifestations

## Abstract

Background: Leukemia is a neoplastic disorder that affects blood and bone marrow and is characterized by white blood cells' abnormal proliferation. Leukemia patients may present with different ophthalmic manifestations. This study aims to provide an updated data about the prevalence and types of ocular manifestations in acute leukemia patients and their relation with the hematological parameters.

Methods: This retrospective cross-sectional study included all acute leukemia patients diagnosed from 2015 to 2020 and underwent an ophthalmic examination during this period at King Abdulaziz Medical City in Jeddah.

Results: Eighty-one patients met the inclusion and exclusion criteria and had ophthalmic examinations. Forty-three (53.1%) patients were males, and 38 (46.9%) patients were females. Acute lymphocytic leukemia (ALL) was diagnosed in 55 (67.9%) patients, while acute myelogenous leukemia (AML) was diagnosed only in 26 (32.1%). Ophthalmic manifestations were observed in 23 patients with a prevalence of 28.4%. AML patients had more manifestations with a rate of 38.5%. ALL had a rate of 23.6% (p=0.1). Retinal hemorrhage was the most commonly seen manifestation in six patients.

Conclusion: Ophthalmic manifestations are not uncommon in acute leukemia patients. Low hemoglobin and RBC could give an idea about the type of ophthalmic manifestation, not the presence or absence. It is highly recommended to examine acute leukemia patients routinely prior, during, and after the treatment to prevent serious ocular damage and monitor the course of the disease.

## Introduction

Leukemia is a neoplastic disorder that affects blood and bone marrow and is characterized by white blood cells' abnormal proliferation [1]. Leukemia is ranked the eleventh in causing death compared to other types of cancers [2]. It is classified upon the duration into acute or chronic [1]. Both are further classified with respect to cell type as either myelogenous or lymphocytic [1,3]. Thus, the diagnosis of leukemia will be either acute myelogenous leukemia (AML), acute lymphocytic leukemia (ALL), chronic myelogenous leukemia (CML), or chronic lymphocytic leukemia (CLL) [1,3].

Bone marrow infiltration in leukemia causes hematological abnormalities such as anemia, thrombocytopenia, or pancytopenia [4,5]. Patients may present with fever, fatigue, bruising, infections, or hepatosplenomegaly [6]. Moreover, ocular infiltration is the third most common extramedullary presentation of acute leukemia after the meninges and testicles [7]. Also, up to 90% of patients with leukemia could present with ocular manifestations during their disease [8]. Ocular manifestations may be caused by the infiltration of circulating leukemic cells in the optic disc, uvea, retinal nerve fibers, and other intraocular tissues [7].

Some leukemia patients may have ocular involvement that could precede the systemic features of the disease [8]. According to different studies, retinal hemorrhage is the most common ophthalmic manifestation in leukemia patients [7-10]. Acute leukemia patients are more prone to ophthalmic manifestations than chronic leukemia [11]. 

Knowledge about the ocular manifestations and their relation to leukemia is not very understood. These manifestations may help in the early diagnosis of systemic leukemia or present as the first sign of an isolated focal relapse following a complete recovery of systemic leukemia [4,10,12]. From 1995 to 1996, three articles studied the ophthalmic manifestations of leukemia patients in Saudi Arabia with a prevalence ranging from 35% to 54%. However, these three studies are quite dated [13-15]. For that reason, this study aims to provide updated data about the prevalence and types of ocular manifestations in acute leukemia patients and their relation with the hematological parameters.

## Materials and methods

This retrospective case-control study included all acute leukemia patients diagnosed from 2015 to 2020 and underwent an ophthalmic examination during this period for any reason at King Abdulaziz Medical City in Jeddah. The study was approved by the Institutional Review Board (IRB) at King Abdullah International Medical Research Center (KAIMRC), IRB reference number: JED-20-427780-13589. Patients with a history of diabetes mellitus, hypertension, or sickle cell disease were excluded. Using a data collection sheet, we collected data from the electronic medical records of the patients. Ophthalmic examination data that included visual acuity, clinical presentation, and the ophthalmic manifestations were collected from ophthalmology department documentation. Ophthalmic manifestations not related to leukemia, such as refractive errors and cataracts, were not considered as positive cases of ophthalmic manifestations. Other data related to the patient's demographics, leukemia type, chemotherapy status, and laboratory hematological findings at the time of examination were also collected. Patients of the ophthalmic manifestation group and the normal ophthalmic examination group were compared. Patients of the ophthalmic manifestation group were further divided into hemorrhagic and non-hemorrhagic manifestations groups to look for differences between the types of ophthalmic manifestations. After data was entered into the datasheet, it was analyzed using Statistical Package for the Social Sciences (SPSS), version 20 (SPSS Inc., Chicago, IL, USA). Categorical variables were reported using frequencies and percentages, while numerical variables were reported as mean and standard deviation (SD) or median and interquartile range (IQR) for skewed data. Groups were compared using chi-square or Fisher's exact tests in regards to qualitative variables. T-test or Mann-Whitney U test was used for the comparison of the quantitative variables between groups. A p-value lower than 0.05 was considered significant.

## Results

After reviewing 270 records of acute leukemia patients, 81 patients met the inclusion and exclusion criteria and had ophthalmic examinations. Forty-three (53.1%) patients were males, and 38 (46.9%) patients were females. ALL was diagnosed in 55 (67.9%) patients, while AML was diagnosed only in 26 (32.1%). Ophthalmic manifestations were observed in 23 patients with a prevalence of 28.4%. Different types of ophthalmic manifestations were reported, with retinal hemorrhage being the most common as shown in Table 1.

**Table 1:** Various types of ophthalmic manifestations among acute leukemia patients. ALL= acute lymphocytic leukemia; AML= acute myelogenous leukemia; CMV= cytomegalovirus.

Type of ophthalmic manifestation		ALL=55 n(%)	AML=26 n(%)	Total=81 n(%)
	No manifestation	42(76.4)	16(61.5)	58(71.6)
	Retinal hemorrhage	3(5.5)	3(11.5)	6(7.4)
	Subconjunctival hemorrhage	2(3.6)	1(3.8)	3(3.7)
	Optic nerve involvement	3(5.5)	-	3(3.7)
	Subhyaloid hemorrhage	1(1.8)	1(3.8)	2(2.5)
	Vitreous hemorrhage	-	2(7.7)	2(2.5)
	Retinal infiltration	2(3.6)	-	2(2.5)
	Splinter hemorrhage	-	1(3.8)	1(1.2)
	Dot hemorrhage	1(1.8)	-	1(1.2)
	Papilledema	-	1(3.8)	1(1.2)
	Papilledema, vitreous infiltration, and retinal detachment	-	1(3.8)	1(1.2)
	CMV retinitis	1(1.8)	-	1(1.2)

**Figure 1:**
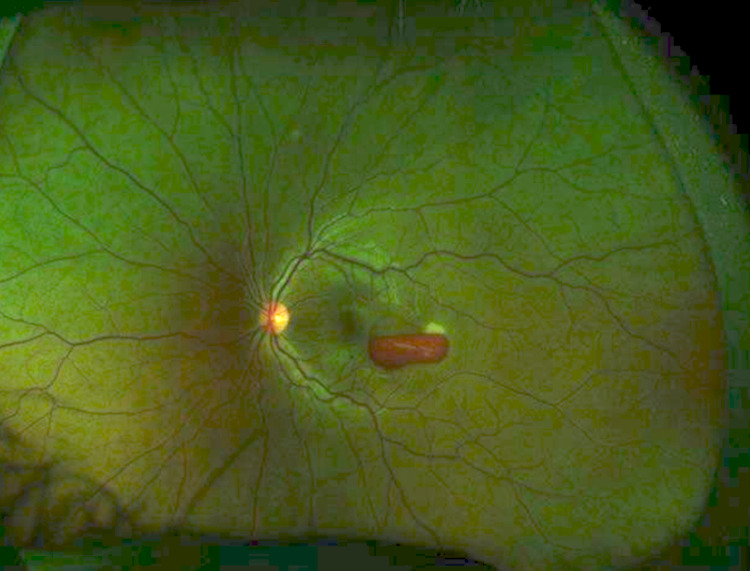
Fundus photograph of AML patient with epi-retinal hemorrhage. AML= acute myelogenous leukemia

An example of ophthalmic manifestation is shown in Figure 1.

AML patients had more manifestations than ALL patients, with a rate of 38.5% and 23.6% (p=0.1), respectively. The presence or absence of ophthalmic manifestations for the 81 patients was compared by gender, type of leukemia, clinical presentation, hematological laboratory findings, and timing of examination in relation to chemotherapy, as shown in Table 2. 

**Table 2:** The difference between the presence and absence of ophthalmic manifestations in regard to different variables. ALL= acute lymphocytic leukemia; AML= acute myelogenous leukemia; WBC= white blood cells; RBC= red blood cells. *For the p-value, the tests used are chi-square test, Fisher's exact test, t-test, and Mann-Whitney U test.

variable	Overall n=81	Ophthalmic manifestation n=23	Normal ophthalmic examination n=58	P value*
Gender				0.5
Male	43	11(25.6%)	32(74.4%)	
Female	38	12(31.6%)	26(68.4%)	
Leukemia type				0.1
ALL	55	13(23.6%)	42(76.4%)	
AML	26	10(38.5%)	16(61.5%)	
Ophthalmic and laboratory findings				
Symptomatic at examination	44	19(43.2%)	25(56.8%)	0.001
Asymptomatic at examination	37	4(10.8%)	33(89.2%)	
WBC at examination Median (IQR)	3.2(3.2)	2.7(6.4)	3.4(3.02)	0.550
RBC at examination Mean (SD)	3.60(0.78)	3.4(0.7)	3.6(0.8)	0.291
Platelets at examination Median (IQR)	119(177)	38(238)	125(130)	0.190
Hemoglobin at examination Median (IQR)	10.1(3)	9.8(2.3)	10.35(3.23)	0.07
Timing of examination				0.6
At diagnosis	9	3(33.3%)	6(66.7%)	
On therapy	42	10(23.8%)	32(76.2%)	
Off therapy	30	10(33.3%)	20(66.7%)	

Out of the 81 patients, there were 20 deceased patients. Nine of the 20 mortality were associated with AML (34.6%), and 11 with ALL (20%) (p=0.154). No association was found between mortality and ophthalmic manifestations (P > 0.05). 

The 23 patients with ophthalmic manifestations were further divided based on the type into hemorrhagic and non-hemorrhagic manifestation groups. Hemorrhagic manifestations were seen in 15 patients, while the non-hemorrhagic were seen in eight patients. Gender, leukemia types, and the hematological laboratory findings at the time of examination for the two groups of ophthalmic manifestations are shown in Table 3.

**Table 3:** The differences between hemorrhagic and non-hemorrhagic ophthalmic manifestations in regard to gender, leukemia types, and hematological parameters. ALL= acute lymphocytic leukemia; AML= acute myelogenous leukemia; WBC= white blood cells; RBC= red blood cells. *For the p-value, the tests used are chi-square test, Fisher's exact test, t-test, and Mann-Whitney U test.

variable	overall n=23	hemorrhagic manifestation n=15	Non-hemorrhagic manifestations n=8	P value
Gender				0.4
Male	11	6(54.5%)	5(45.5%)	
Female	12	9(75%)	3(25%)	
Leukemia type				0.19
ALL	13	7(53.8%)	6(46.2%)	
AML	10	8(80%)	2(20%)	
WBC Median (IQR)	2.7(6.4)	1.9(2.6)	5.3(5.5)	0.06
RBC Mean (SD)	3.4(0.72)	3.2(0.6)	3.8(0.77)	0.04
Platelets Median (IQR)	38(238)	31(115)	227(273.5)	0.09
Hemoglobin Mean (SD)	9.7(1.8)	9.5(1.5)	11.08(1.75)	0.009

Patients who had ophthalmic manifestations were also divided based on the timing of ophthalmic examination. Three ophthalmic manifestations were seen at the time of diagnosis before the initiation of therapy, ten were during therapy, and the other ten were after therapy. The characteristics of the three patients who had the manifestations early before chemotherapy are shown in Table 4.

**Table 4:** Characteristics of the three ophthalmic manifestations identified at the time of leukemia diagnosis. ALL= acute lymphocytic leukemia; AML= acute myelogenous leukemia; WBC= white blood cells; RBC= red blood cells.

Patient serial number	age	gender	Type of leukemia	Ophthalmic manifestation	WBC	RBC	Platelets	Hemoglobin
Patient 1	39	Male	AML	Vitreous hemorrhage	129.7	2.6	18	7
Patient 2	5	Female	ALL	retinal hemorrhage	1.1	4.2	35	12.3
Patient 3	4	Male	ALL	retinal hemorrhage	0.8	2.8	37	8.2

Patients who were examined during the therapy had a higher rate of hemorrhagic manifestations (21.4%) compared to the off therapy patients (10%), and a higher rate of non-hemorrhagic manifestations (23.3%) was associated with the off therapy patients compared with patients on therapy (2.4%) (p=0.014). Of the ten manifestations that were detected during active treatment of chemotherapy, nine of them were hemorrhagic. Also, seven of the manifestations were after the second cycle of the chemotherapy. However, only three manifestations were detected after the first cycle with various characteristics as shown in Table 5.

**Table 5:** Characteristics of the ophthalmic manifestations diagnosed after the first cycle of treatment. AML= acute myelogenous leukemia; WBC= white blood cells; RBC= red blood cells.

Patient serial number	Leukemia type	Ophthalmic manifestation	WBC	RBC	Platelets	Hemoglobin
Patient 1	AML	papilledema, vitreous infiltration, and retinal detachment	7.5	3.9	402	10.9
Patient 2	AML	vitreous hemorrhage	0.6	2.6	31	7.5
Patient 3	AML	splinter hemorrhage	0.9	3.1	18	8.6

For the ten patients examined after stopping chemotherapy, seven manifestations were non-hemorrhagic, and three were hemorrhagic manifestations. While most of the patients were examined late after stopping chemotherapy, three manifestations were detected early during the first month of stopping chemotherapy with their characteristics being described in Table 6.

**Table 6:** Characteristics of the ophthalmic manifestations diagnosed within the first month after stopping the treatments. ALL= acute lymphocytic leukemia; AML= acute myelogenous leukemia; WBC= white blood cells; RBC= red blood cells.

Patient serial number	Leukemia type	Ophthalmic manifestation	WBC	RBC	Platelets	Hemoglobin
Patient 1	ALL	optic nerve involvement	3	3.5	10	10.3
Patient 2	ALL	retinal hemorrhage	0.1	2.6	11	7.7
Patient 3	AML	splinter hemorrhage	0.4	3.4	44	9.7

## Discussion

Ophthalmic manifestations have been reported in the literature with different prevalence from study to study, ranging from 24% to 70% [4,8-10,13-17]. Also, some studies that have been done in Saudi Arabia reported prevalence ranging from 35%-54%; however, these studies are old and published in the '90s [13-15]. This wide range of reported prevalence could be due to differences in ethnicity of the study population, study design, criteria of inclusion and exclusion, or timing of ophthalmic manifestations diagnosis in relation to leukemia. This study has identified the presence of various ophthalmic manifestations in 23 acute leukemia patients with a prevalence of 28.4%. Retinal hemorrhage was the most commonly seen among the 23 manifestations, but it was not associated with a specific type of acute leukemia. Similar to this study, most studies have reported retinal hemorrhage as the most common ophthalmic manifestation in acute leukemia patients [4,5,8-10]. 

The study did not show any statistical difference regarding the presence or absence of ophthalmic manifestations for the ALL and AML groups; however, AML carried a higher rate of manifestation than the ALL group. This higher rate of ophthalmic manifestations in AML leukemia has been reported in many studies [4,5,9,10,16]. Moreover, in this study, some serious manifestations were seen only in AML patients. These manifestations include vitreous hemorrhage, papilledema, vitreous infiltration, and retinal detachment. Also, looking at the six manifestations identified at diagnosis and early after the first cycle of therapy, four of these six early manifestations were associated with AML. These data may suggest that AML patients are more prone to early and serious ophthalmic manifestations than ALL patients.

Ophthalmic findings are common in patients who have vision complaints but even asymptomatic patients can have undiscovered ophthalmic findings. Only four (17.4%) of the ophthalmic manifestations were discovered incidentally with no clinical symptoms or complaints. This is unlike what was reported by Bitirgen et al. as their asymptomatic clinical manifestations were seen in 62.2% of the manifestations, and to what was reported by Hafeez et al. as 70% of the ophthalmic manifestations were asymptomatic [4,10]. This could be due to the fact that out of the 270 acute leukemia patients who were diagnosed in our hospital, only 81 patients had ophthalmic examinations, and to the fact that all the examinations were done because of the clinical complaints of the patients or few patients because of the routine examination before bone marrow transplant. 

The majority of ophthalmic manifestations in leukemia patients are not due to the direct infiltration of the disease but mostly due to the changes in hematological parameters which may cause bleeding [5]. Some studies have discussed the association between the ophthalmic manifestations and blood parameters [4,5,15]. The high platelet count was associated with a reduction in the overall incidence of ophthalmic manifestations, and a reduction in hemoglobin was more associated with subhyaloid hemorrhage as reported by Soman et al. [5]. Another study by Bitirgen et al. also reported the significant association of the low platelet count with ophthalmic manifestations [4]. Moreover, retinal hemorrhage was seen more in anemic patients in the study by Abu El-Asrar et al. [15]. In this study, the overall presence or absence of ophthalmic manifestations showed no difference regarding WBC, RBC, platelets, and hemoglobin count. However, the blood parameters could hint at differentiating the type of the ophthalmic manifestations because hemorrhagic manifestations were associated with low hemoglobin and RBC mean (which could be due to bone marrow suppression or infiltration) compared to the non-hemorrhagic manifestations. 

The course of the disease and timing of the ophthalmic examination could affect the type of manifestation in this study. Hemorrhagic manifestation came usually in early phases of diagnosis which could be due to the bone marrow suppression by leukemia or by treatment. The period after stopping the therapy, especially after one month of stopping the therapy, was associated more with the non-hemorrhagic manifestations, which could be a sign of relapse. 

This study has some limitations. First, it is a retrospective cross-sectional study that includes a small number of leukemia patients, which may not reflect the true prevalence of ophthalmic manifestations in our population because leukemia patients are not undergoing ophthalmic examinations routinely. Second, the effects of the disease itself and leukemia treatment are overlapping and indistinguishable.

## Conclusions

From the study results, ophthalmic manifestations are not uncommon in acute leukemia patients, with retinal hemorrhage being the most commonly seen manifestation. Low hemoglobin and RBC could give an idea about the type of ophthalmic manifestation, not the presence or absence. Although it is very difficult to make such a conclusion, it is highly recommended to examine acute leukemia patients prior, during, and after the treatment to prevent serious ocular damage and monitor the course of the disease.
